# Peritumoral Immune Infiltrate as a Prognostic Biomarker in Thin Melanoma

**DOI:** 10.3389/fimmu.2020.561390

**Published:** 2020-09-29

**Authors:** Francesco Sabbatino, Giosuè Scognamiglio, Luigi Liguori, Antonio Marra, Anna Maria Anniciello, Giovanna Polcaro, Jessica Dal Col, Alessandro Caputo, Anna Lucia Peluso, Gerardo Botti, Pio Zeppa, Soldano Ferrone, Stefano Pepe

**Affiliations:** ^1^ Department of Medicine, Surgery and Dentistry “Scuola Medica Salernitana”, University of Salerno, Baronissi, Italy; ^2^ Oncology Unit, San Giovanni di Dio e Ruggi D’Aragona University Hospital, Salerno, Italy; ^3^ Pathology Unit, Istituto Nazionale Tumori, IRCSS, “Fondazione G. Pascale”, Naples, Italy; ^4^ Department of Clinical Medicine and Surgery, University of Naples “Federico II”, Naples, Italy; ^5^ Division of Early Drug Development for Innovative Therapies, IEO, European Institute of Oncology, Milan, Italy; ^6^ Pathology Unit, San Giovanni di Dio e Ruggi D’Aragona University Hospital, Salerno, Italy; ^7^ Scientific Direction, Istituto Nazionale Tumori-IRCCS-Fondazione G. Pascale, Naples, Italy; ^8^ Division of Surgical Oncology, Department of Surgery, Massachusetts General Hospital, Harvard Medical School, Boston, MA, United States

**Keywords:** ****thin melanoma, ****tumor-infiltrating lymphocytes, CD4, CD8, time, human leukocyte antigen class I antigens, programmed death-ligand 1, prognosis

## Abstract

Thin melanomas are tumors less than 1 mm thick according to Breslow classification. Their prognosis is in most cases excellent. However, a small subset of these tumors relapses. These clinical findings emphasize the need of novel prognostic biomarkers to identify this subset of tumors. Characterization of tumor immune microenvironment (TIME) is currently investigated as a prognostic and predictive biomarker for cancer immunotherapy in several solid tumors including melanoma. Here, taking into account the limited availability of tumor tissues, by characterizing some of the characteristics of TIME such as number of infiltrating lymphocytes, HLA class I antigen and PD-L1 expression, we show that number of infiltrating CD8+ and FOXP3+ T cells as well as CD8+/FOXP3+ T cell ratio can represent a useful prognostic biomarker in thin melanoma. Although further investigations in a larger patient cohort are needed, these findings have potential clinical significance since they can be used to define subgroups of thin melanoma patients who have a worse prognosis and might need different treatment modalities.

## Introduction

The incidence of skin melanoma is increasing worldwide with 60.7 per 100,000 patients dead for this type of cancer in 2018. (1). Considerable disparities in terms of survival exist between loco-regional and metastatic melanoma with 5-year relative survival rates of 64–98% and 20%, respectively (2), although recent advances in targeted therapy and immunotherapy have dramatically improved metastatic melanoma patients’ overall survival. Hopefully, most of new melanomas are diagnosed at an early stage and up to one third of new cases are detected as *in-situ* lesions (2). According to TNM classification, 5- and 10-year survival rates are different within the T subgroups, ranging from 97 to 93% and from 53 to 39% for patients with T1 (Breslow depth <1 mm)—usually termed thin melanoma—and T4 melanomas, respectively (3). In order to better define thin melanoma prognosis, the presence of aggressive features such as depth higher than 0.75/0.85 mm and presence of ulceration are currently utilized as major prognostic biomarkers (4, 5). In addition, other characteristics of thin melanoma, including lymphovascular invasion and tumor regression, have been also associated with a higher risk of recurrence (6). Nevertheless, some thin melanomas with such favorable prognostic features, develop local or distant metastases. These clinical findings emphasize the need to integrate more effective prognostic biomarkers in those clinicopathological characteristics. Several lines of evidence have highlighted the crucial role of tumor immune microenvironment (TIME) in melanoma biology (7, 8). Overall the presence of tumor-infiltrating lymphocytes (TILs) in melanomas has been associated with a better patient prognosis, regardless of other clinicopathological characteristics (9–15), although the presence of TILs in improving melanoma prognosis remains controversial (16). In addition, both characterization of the tumor immune infiltrate by CD8+ and regulatory CD4+ T cells and detection of tumor immune escape mechanisms, including programmed death-ligand 1 (PD-L1) up-regulation and human leukocyte antigen (HLA) class I down-regulation on cancer and/or immune cells, have been shown to better define the melanoma disease course (17–23). However, scant data about the prognostic role of TIME characteristics in thin melanoma subgroups are available so far. Therefore, the aim of our study was to analyze some of the TIME characteristics in a cohort of patients with surgically resected thin melanoma and to evaluate their potential clinical significance.

## Materials and Methods

### Patients and Tumor Tissues

Formalin-fixed paraffin-embedded (FFPE) tumor specimens were obtained from patients with surgically resected thin melanoma (depth ≤ 1 mm) followed at “San Giovanni di Dio e Ruggi D’Aragona” University Hospital between 2010 and 2020. Patient selection was performed based on tumor tissue and clinical record availability. All samples were from Caucasian patients. Patients were consented for tissue acquisition per institutional review board-approved protocol. All patients signed an informed consent. Diagnosis of melanoma was confirmed by an independent, skin cancer-dedicated pathologist (PZ). Presence of tumor cells in FFPE was monitored by hematoxylin and eosin staining. Melanomas were staged according to the 8^th^ Edition of the American Joint Committee on Cancer (AJCC) classification system. Sentinel lymph node biopsy was performed in all patients if melanoma lesion was > 0.75mm thick or in presence of ulceration. Patient demographic (age and gender), pathological (TNM stage, Breslow depth, tumor localization, presence of ulceration and/or regression, number of mitosis/mm^2^, presence of TILs) and disease free survival (DFS) data were retrieved from clinical records. Presence of regression was defined as partial, segmental or complete replacement of melanoma cells with a variable host response including dense mononuclear infiltrate, melanophages and/or dermal fibrosis accompanied by increased dermal vascularity, with variable epidermal attenuation (24–26). In addition to further characterize the prognostic role of tumor regression a 10% cut off of regression extension was also adopted as previously reported (27).

### Monoclonal Antibodies

The monoclonal antibodies (mAbs) HCA2, which recognizes β2m-free HLA-A (excluding -A24), -B7301, and -G heavy chains (28, 29), and HC10, which recognizes β2m-free HLA-A3, -A10, -A28, -A29, -A30, -A31, -A32, -A33, and all β2m-free -HLA-B (excluding -B5702, -B5804, and -B73) and -HLA-C heavy chains (28–30), were developed and characterized as previously described. mAbs were purified from ascitic fluid by affinity chromatography on a Protein G column. Purity and activity of mAb preparations were monitored by SDS-PAGE and by specific reactivity with the corresponding antigen in binding assays and Western blotting. The CD8-specific mAb C8/144B (Dako), the FOXP3-specific mAb D2W8E (Cell Signaling), the Granzyme B (GRZ-B)-specific mAb ab4059 (Abcam) and the PD-L1-specific mAb E1L3N (Cell Signaling) were purchased from the indicated companies. The mouse mAb E7Q5L (IgG2b) was used as an isotype control for HCA2 and HC10 staining. The rabbit mAb DA1E (IgG) was used as an isotype control for PD-L1 staining.

### Immunohistochemical Staining

FFPE tissue sections (4 μm) from melanoma tumor samples were used as substrates in immunohistochemical (IHC) reactions. IHC staining with a pool of mAb HCA2 and HC10 (in a 1:1 ratio) was performed and scored as described previously (31–33). The staining with C8/144B, D2W8E, ab4059 and E1L3N mAbs was performed on BOND automated IHC Stainer (Leica). The double staining with C8/144B and D2W8E mAbs was performed utilizing the ChromoPlex 1 Dual Detection kit (Leica) while the staining with ab4059 and E1L3N mAbs was performed utilizing 3,3’ diaminobenzidine (DAB) chromogen (Dako). Staining intensity and percentage of stained tumor cells in each lesion were reviewed and enumerated by two experienced pathologists (AA and PZ) who had no knowledge of the patients’ characteristics and clinical outcomes. Staining with CD8-, FOXP3-, GRZ-B- and PD-L1-specific mAbs was performed according to the manufacturers’ instructions. Staining with CD8-, FOXP3-, GRZ-B- and PD-L1-specific mAbs was scored by counting the number of stained cells in four high-powered fields of melanoma lesions. For CD8-, FOXP3-, GRZ-B- scoring both peritumoral and intratumoral positive cells were counted. Peritumoral positive cells were defined as cells distributed along the stromal–tumor interface at tumor edge in dermis. Intratumoral positive cells were defined as cells completely surrounded the neoplastic cells. For scoring of PD-L1 staining, only tumor samples demonstrating cell membrane staining were considered positive. PD-L1 expression was evaluated only on tumor cells.

### Statistical Analysis

Statistical analysis was performed with the Stata Statistical Software, Version 13.0 (StataCorp,LP). Correlation of HLA class I antigen and PD-L1 expression with the number of CD8+, FOXP3+ and GRZ-B+ T cells was analyzed by Spearman rank correlation test. Correlation of PD-L1 expression with HLA class I antigen expression was analyzed by Fisher exact test. Differences in the expression levels of variables according to histopathologic and clinical characteristics were analyzed using the Mann–Whitney U test or the Kruskal–Wallis rank test. Disease-free survival (DFS) was calculated using the Kaplan–Meier method. Time was defined as the interval between the date of diagnosis and the date of disease recurrence (event) or that of last follow-up visit (censored). The log-rank test was used for screening the potential prognostic factors in relation to patients’ survival. p<0.05 was considered to be statistically significant. All tests used were two-tailed.

## Results

### Patient Characteristics

**Figure 1:**
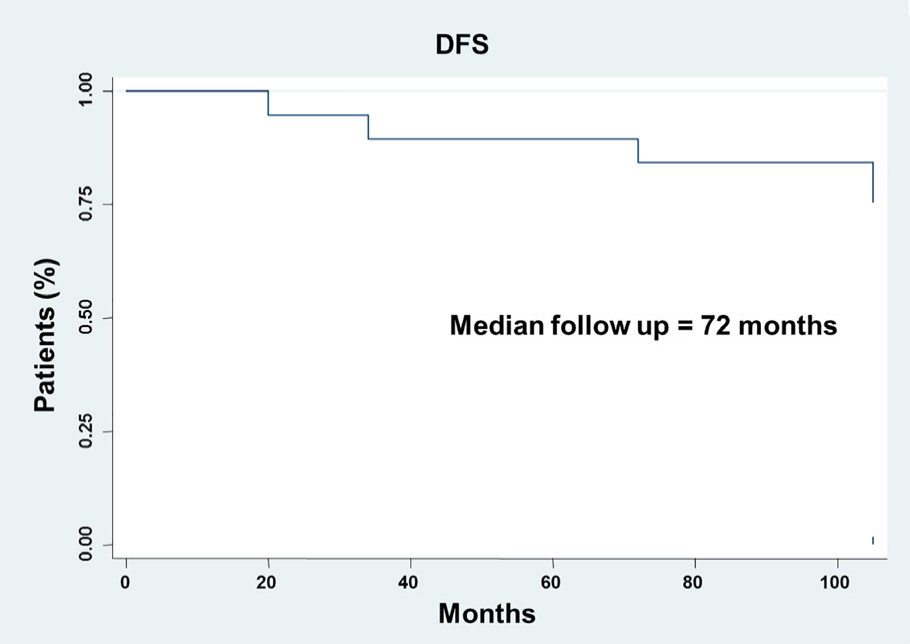
Analysis of the disease-free survival (DFS) in thin melanoma. DFS analysis was analyzed using the Kaplan-Meier method.

Thirty-one patients were included in the study. Patient and tumor characteristics are summarized in **Table 1**. Median age was 56.4 years (range, 30–78). Eighteen patients (58.1%) were female. Primary tumor localization was head/neck, limbs and trunk in 16.1, 38.7, and 38.7% of patients, respectively. The mean Breslow depth was 0.4 mm (0.1–1.0 mm). Ulceration was present in 3 (9.7%) out of the 31 patients investigated. Regression was present in 64.5% of the samples analyzed. Adopting a 10% cut off of extension, regression was present in 41.9% of sample analyzed. Twenty-three melanomas were classified as T1a (74.2%) and 8 as T1b (25.8%). The mean number of mitosis was 1.3 mm^2^ (0.0–4.0). TILs were present in 13 melanomas (42.0%), defined as absent in 9 (29.0%) and not reported in 9 (29.0%). Metastases were not detected in all sentinel lymph node biopsies analyzed. At a median follow up of 92.0 months (20.0-105.0 months), the median DFS was not reached (**Figure 1**). Four (12.9%) out of the 31 patients investigated developed a distant recurrence during the follow up.

**Table 1:** Patient and tumor characteristics.

Age	56.4 (30–78)
Gender	
Male	13 (41.9%)
Female	18 (58.1%)
Anatomic site	
Head/neck	5 (16.1%)
Limbs	12 (38.7%)
Trunk	12 (38.7%)
Not evaluable	2 (6.5%)
Breslow depth	0.4mm (0.1-1.0)
Ulceration	
No	28 (90.3%)
Yes	3 (9.7%)
Regression	
No	2 (6.5%)
Yes	20 (64.5%)
Not evaluable	9 (29.0%)
Regression at 10% cut off	
No	9 (29.0%)
Yes	13 (41.9%)
Not evaluable	9 (29.0%)
TNM^†^	
T1a	23 (74.2%)
T1b	8 (25.8%)
Mitosis^‡^	1.3 (0–4.0)
Tumor-infiltrating lymphocytes	
Absent	9 (29.0%)
Present	13 (42.0%)
Not evaluable	9 (29.0%)

^†^Staging according to the 8^th^Edition of AJCC melanoma classification.

^‡^Number of mitosis per square millimeter.

### Correlation Between Clinical and Pathological Characteristics in Thin Melanomas

**Figure 2:**
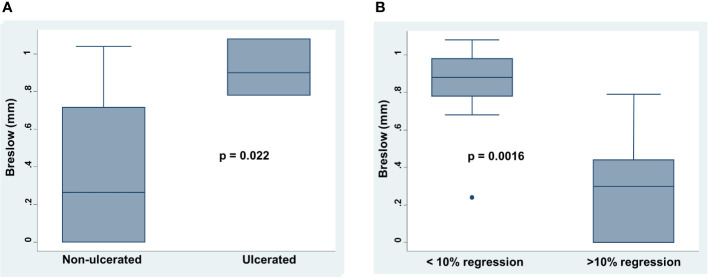
Correlation between Breslow depth , presence of ulceration and tumor regression in thin melanoma. **(A)** Presence or absence of ulceration was correlated to Breslow depth by Mann-Whitney U test. **(B)** Presence or absence of regression at 10% cut off of extension was correlated to Breslow depth by Mann-Whitney U test. On each box, the central mark is the median, the edges of the box are the 25^th^ and 75^th^ percentiles, the whiskers extend to the most extreme data points note considered outliers, and outliers are plotted individually. p was considered significant if < 0.05.

Breslow depth positively correlated with the presence of ulceration (p=0.022). Ulcerated melanomas had an increased depth as compared to that of non-ulcerated (mean 0.92 versus 0.35 mm; **Figure 2A**). In addition, Breslow depth positively correlated with the number of mitosis (Spearman’s rho 0.5004, p=0.021). Lastly, less than 10% of regression positively correlated with high Breslow (mean 0.82 versus 0.32 mm p= 0.0016) and presence of ulceration (p= 0.055) (**Figure 2B**).

### Correlation of Immune Cell Infiltrate With Clinicopathological Characteristics of Thin Melanoma Tumors

**Figure 3:**
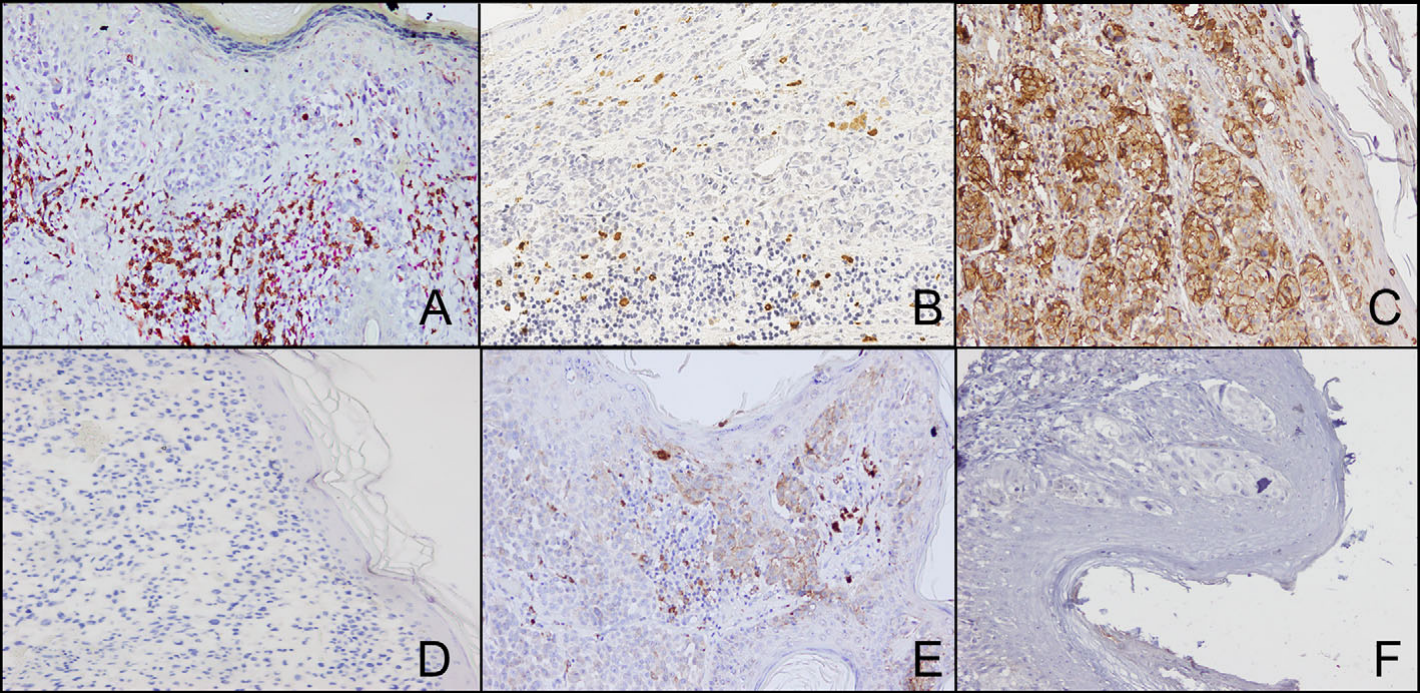
Representative staining patterns of formalin-fixed, paraffin-embedded thin melanoma lesions with CD8- **(A)**, FOXP3- **(A)**, GRZ-B- **(B)**, HLA class I antigen- **(C)** and PD-L1- **(E)** specific mAbs. Double IHC staining was performed utilizing CD8- (brown cells) and FOXP3- (red cells) specific mAbs **(A)**. For HLA class I antigen detection tissue sections were IHC stained with a pool of mouse HLA-A–specific mAb HCA2 and HLA-B/C-specific mAb HC10 (ratio, 1:1). mAbs E7Q5L (IgG2b) and DA1E (IgG) were used as isotype controls for HLA class I antigen **(D)** and PD-L1 **(F)** staining, respectively. Magnification is 200X.

Representative IHC staining of CD8+, FOXP3+ and GRZ-B+ T cells is shown in **Figures 3A, B**. The number of peritumoral CD8+ and GRZ-B+ T cells ranged from 4 to 89 (mean 48.3) and from 0 to 12 (mean 4.5), respectively. The number of intratumoral CD8+ and GRZ-B+ T cells ranged from 1 to 14 (mean 7.6) and from 0 to 2 (mean 0.58), respectively. On the other hand, the number of peritumoral and intratumoral FOXP3+ T cells ranged from 0 to 87 (mean 19.4) and from 0 to 13 (mean 3.0), respectively. CD8+/FOXP3+ T cell ratio ranged from 0.6 to 68.0 (mean 9.2). The number of peritumoral CD8+ T cells was positively correlated with number of intratumoral CD8+ T cells (Spearman’s rho=0.9892; p<0.0001). The number of peritumoral FOXP3+ T cells was positively correlated with number of intratumoral FOXP3+ T cells (Spearman’s rho=0.9472; p<0.0001). The number of peritumoral and intratumoral CD8+ T cells was positively correlated with the number of peritumoral (Spearman’s rho=0.40004; p=0.031) and intratumoral (Spearman’s rho=0.43; p=0.019) FOXP3+ T cells, respectively.

Representative IHC staining of HLA class I antigen expression by thin melanoma cells is shown in **Figures 3C, D**. HLA class I antigen expression was down-regulated in 5 (16.1%) tumor samples but was within normal ranges in the remaining 24 (77.4%). HLA class I antigen expression ranged from 0 to 70 (mean 28.7).

**Figure 4:**
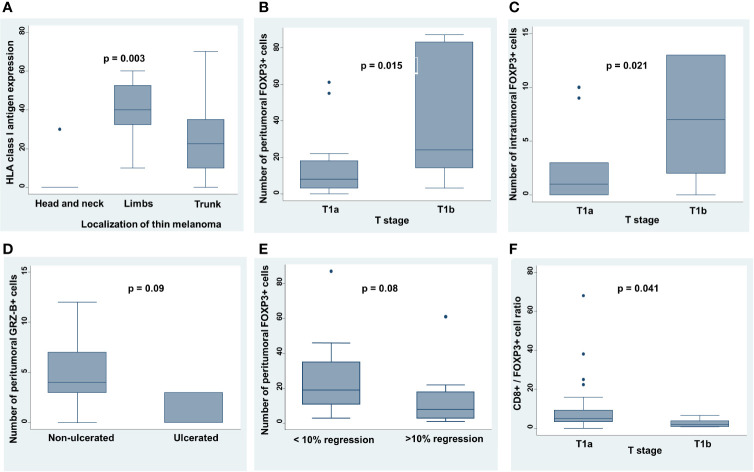
Correlation between clinicopathological features and TIME characteristics in thin melanoma. **(A)** Site of primary thin melanoma was correlated to HLA class I antigen expression level by Kruskal-Wallis rank test. **(B, C)** T stage (sec. TNM, AJCC 8^th^ Edition) was correlated to the number of peritumoral and intratumoral FOXP3+ T cells by Mann-Whitney U test. **(D)** Presence or absence of ulceration was correlated to the number of peritumoral GRZ-B+ T cells by Mann-Whitney U test. **(E)** Number of peritumoral FOXP3+ T cells was correlated with presence or absence of regression at 10% cut off of extension by Mann-Whitney U test. **(F)** T stage (sec. TNM, AJCC 8^th^ Edition) was correlated to CD8+/FOXP3+ T cell ratio by Mann-Whitney U test. On each box, the central mark is the median, the edges of the box are the 25^th^ and 75^th^ percentiles, the whiskers extend to the most extreme data points note considered outliers, and outliers are plotted individually. p was considered significant if < 0.05.

Melanomas localized to head/neck and trunk were associated with a higher level of TILs as compared to those to limbs (p=0.017). In addition, melanomas of limbs expressed a higher HLA class I antigen level as compared to those localized to trunk and to head and neck (median 40.8, 25.5 and 6.0, respectively; p=0.003) (**Figure 4A**). Presence of TILs positively correlated with presence of more than 10% of regression extension (p=0.020).

The number of peritumoral and intratumoral FOXP3+ T cells in dermis was positively correlated with T stage subgroups (sec. TNM, AJCC 8^th^ Edition) (peritumoral p=0.015; **Figure 4B** **)** (intratumoral p=0.021; **Figure 4C**). In addition, ulcerated tumors had a lower number of peritumoral GRZ-B+ T cells as compared to that of non-ulcerated tumors (p=0.09; **Figure 4D**) and tumors with less than 10% of regression had a higher number of peritumoral FOXP3+ T cells as compared to that of more than 10% (p=0.08; **Figure 4E**). Lastly, CD8+/FOXP3+ T cell ratio negatively correlated with T stage subgroups (sec. TNM, AJCC 8^th^ Edition) (p=0.041; **Figure 4F**).

### PD-L1 Expression in Thin Melanomas and Its Interaction With Immune Infiltrate

Representative IHC staining of PD-L1 expression by thin melanoma cells is shown in **Figures 3E, F**. PD-L1 expression was detected in 10 (32.3%) out of 31 samples analyzed. The mean number of PD-L1+ cells was 3.29 per high powered field (0.0–25.0). No significant correlations were found between PD-L1 expression and the other clinicopathological characteristics of the samples analyzed as well as between PD-L1 expression and immune infiltrate.

### Correlation of TIME and Clinicopathological Characteristics With DFS

**Figure 5:**
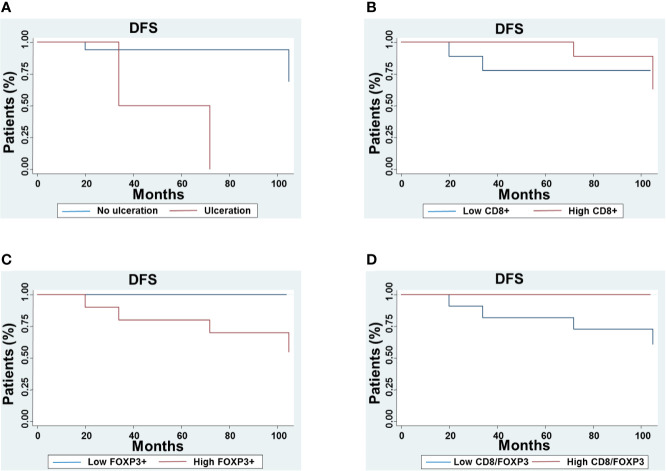
Correlation between disease-free survival (DFS) and TIME characteristics in thin melanoma. The DFS of patients with lesions grouped based on ulceration **(A)**, number of peritumoral CD8+ T cells **(B)**, FOXP3+ cells **(C)** and CD8+/FOXP3+ T cell ratio **(D)** was compared using the Kaplan–Meier method.

At univariate analysis, age (p=0.005), Breslow depth (p=0.005), presence of ulceration (p<0.001), number of mitosis (p=0.026), less than 10% of regression extension (p=0.08) as well as number of peritumoral FOXP3+ T cells (peritumoral p=0.006) were negatively correlated to DFS. In contrast, number of CD8+ T cells (peritumoral p=0.034; intratumoral p=0.005) and CD8+/FOXP3+ T cell ratio (p=0.022) were positively correlated to DFS (**Figure 5**).

## Discussion

Patients with thin melanoma have an excellent prognosis with 10-year survival outcome ranging from 85 to 99% (3). For thin melanoma as well as for thicker melanoma, presence of ulceration, Breslow depth, mitotic rate, lymphovascular invasion and presence of regression are the most relevant prognostic biomarkers (4–6). However, so far, about 10% of surgically removed thin melanomas relapse. Informative biomarkers are needed to improve the stratification of patient prognosis and to design novel therapeutic strategies to prevent tumor relapse in this setting. In the present study, analysis of patient prognosis shows that ulceration, Breslow depth, mitotic rate and less than 10% of regression extension significantly correlate with DFS in thin melanoma. In addition, a high Breslow depth positively correlates with a high number of mitosis, presence of ulceration and less than 10% of regression extension. By corroborating the information in the literature (27, 34, 35) these results validate the representativeness of patient population analyzed in spite of its limited number.

In a recent study, Dessinioti et al. showed that nodular melanomas are associated with a poor prognosis in T1 stage subgroup as compared to that of superficial spreading melanomas (36). We were not able to provide this type of information since in our medical records, melanoma subtypes were not specified. Lundgren et al. demonstrated that a high expression of Kin of IRRE protein (KIRREL) is significantly associated with several unfavourable clinicopathological characteristics, including high Clark level and Breslow depth, presence of ulceration, advanced clinical stage and high mitotic rate in thin melanoma (37). The results of our study suggest that some of TIME characteristics might be useful prognostic biomarkers in patients with thin melanoma.

TIME characteristics are currently investigated as a prognostic and predictive biomarker for cancer immunotherapy in several types of solid cancers including melanoma (38). They play a major role in the host immune response to tumor antigens expressed on melanoma cells (7, 39). The crucial role of host immune response in melanoma is confirmed by the clinical efficacy of immune checkpoint inhibitor-based immunotherapy in this type of tumor (40). Indeed, treatment with anti-Cytotoxic T-Lymphocyte Antigen 4 and anti-Programmed cell Death 1 have dramatically changed the prognosis of melanoma patients (41–45). In this study, because of the limited availability of thin melanoma tissues we could not analyze multiple marker expression in order to fully characterize melanoma thin TIME. We focused our analysis on the characterization of TILs as a marker of host immune response to tumor antigens and HLA class I antigen down-regulation and PD-L1 expression as mechanisms of immune escape by malignant cells.

TILs have been shown to play a favorable prognostic role in melanoma (9–15, 46, 47) as their presence correlates with both Breslow depth and 10 year survival (48). However, the association of TILs with an improved prognosis in melanoma remains controversial. In addition, the impact of different components of TILs on melanoma’s characteristics and prognosis is still under investigation (18, 21, 22). In a recent meta-analysis Fu et al. have confirmed the favorable prognostic role of the CD3+, CD4+, CD8+ TILs in melanoma patients’ overall survival as well as the association between TIL presence and improved overall survival (49). In the present study characterization of some TIME features has demonstrated that both numbers of peritumoral and intratumoral FOXP3+ and CD8+ T cells as well as their ratio might be useful prognostic biomarkers in thin melanomas. Specifically, either a low number of peritumoral or intratumoral CD8+ T cells and a high number of peritumoral FOXP3+ T cells negatively correlate with DFS. Our findings, in thin melanomas, are in line with the previous reports available in the literature for melanoma tumors. De Panfilis et al. demonstrated that number of FOXP3+ lymphocytes is higher in late stage melanoma as compared to that in early stage (17). Miracco et al. demonstrated that a high number of both FOXP3+ and CD25+ T cells in primary tumors correlates with a high rate of tumor relapse in melanoma (50). On the other hand, a high number of TCF7/CD8+ T cells in melanoma tumors has been associated with clinical responses to immunotherapy (51, 52). Furthermore tissue-resident memory CD8+ T cells play a crucial role in the surveillance of subclinical melanoma (23). In our study the number of CD8+ T cells positively correlates with the number of FOXP3+ T cells. In addition, when both CD8+ T cells and FOXP3+ T cells are present in a tumor, they are spatially very close. These findings suggest a potential interaction between CD8+ and FOXP3+ T cells as a mechanism of immune regulation in thin melanoma progression. However, elucidation of the significance of interactions between CD8+ T cells and FOXP3+ T cells requires additional investigations.

In the present study we analyzed the CD8+ and FOXP3+ T cells localized along the stromal–tumor interface at tumor edge in dermis (peritumoral) and those completely surrounding the neoplastic cells (intratumoral). The number of CD8+ and FOXP3+ T cells were positively correlated with number of intratumoral CD8+ and FOXP3+ T cells, respectively. In some cases the number of intratumoral TILs was very low or negative. This was most likely caused by the low size of tumors analyzed. Nevertheless, in almost all cases, their number still correlated with the clinicopathological characteristics of tumors analyzed. Future studies in a larger patient cohort will be needed to validate their potential prognostic role in thin melanoma.

Besides CD8+ and FOXP3+ T cells, we also analysed the number of GRZ-B+ T cells as well as its correlation with clinicopathological characteristics of thin melanoma. We used GRZ-B expression as a readout for CD8+ T-cell cytotoxicity. However double immunofluorescent staining for CD8+ and GRZ-B+ cells demonstrated that GRZ-B did not co-localized with CD8 (**Supplementary Data**). Further studies are needed to characterize the subpopulation of GRZ-B+ cells. Their number showed a negative association, although non statistically significant, with presence of ulceration. The latter is a major unfavourable prognostic biomarker (53, 54). We also analyzed PD-L1 up-regulation and HLA class I down-regulation on cancer cells since both represent two of the major tumor immune escape mechanisms (17, 19, 20, 23). HLA class I antigen and PD-L1 expression are known to play a key role in the host immune response to tumor antigens, since the former is crucial for processing tumor antigens and presentation of tumor antigen derived peptides to cognate T cells, while the latter inhibits T cell activation (19, 20, 53, 55). In our study no significant correlation was found between HLA class I antigen expression level and prognosis in thin melanoma, although HLA class I antigen expression is correlated with thin melanoma localization. Specifically, HLA class I antigen expression level was significantly higher in melanomas localized to limbs as compared to melanomas localized to head and neck or to trunk. The latter expressed the lowest levels of HLA class I antigens. It is well known that localization of primary melanoma is an important prognostic factor with the worst prognosis for melanoma localized to head and neck (56). On the other hand, PD-L1 expression level did not correlate to any of the clinicopathological characteristics of thin melanomas analyzed as well as to DFS. Conflicting data are reported about the prognostic role of PD-L1 expression in melanoma (19). These findings might reflect the lack of standardization of the IHC assay used to measure PD-L1 expression as well as the different characteristics of the PD-L1-specific antibodies utilized to in the assays. PD-L1 is also reported to be expressed on tumor associated macrophages in melanoma tumors (57). However, as we have already discussed above because of limited availability of tissue samples we were not able to analyze more biomarkers including those for tumor associated macrophages. Further studies are needed to investigate the role of tumor associated macrophages as well as the PD-L1 expression on tumor associated macrophages in thin melanomas.

## Conclusions

In conclusion in this study we have shown that some of TIME characteristics may be a useful biomarker to improve thin melanoma prognosis. The low number of patients and tissue samples included in the analysis represents the major limitation of the present study. However, these findings, especially when independently validated in a larger patient cohort, have potential clinical significance since they can be used to define subgroups of thin melanoma patients who might benefit from different treatment modalities. Specifically, they can be useful to design novel clinical trials which utilize immune checkpoint inhibitor-based strategies for the treatment of thin melanoma patients with poor prognosis.

## Data Availability Statement

The raw data supporting the conclusions of this article will be made available by the authors, without undue reservation.

## Ethics Statement

The studies involving human participants were reviewed and approved by ASL Napoli 3 sud Servizio Coordinamento Etico Campania Sud. The patients/participants provided their written informed consent to participate in this study.

## Author Contributions

Conception and design: FS, SF, SP. Development of methodology: FS, GS, SF. Acquisition of data: GS, AA, PZ. Analysis and interpretation of data: FS, GS, AA, PZ, JD. Writing, review, and/or revision of the manuscript: FS, LL, AM, SF. Administrative, technical, or material support (i.e., reporting or organizing data, constructing databases): GP, AC, AP. Study supervision: SP.

Other (contributed clinical and pathological material; discussed results and implications of findings): GB, SF, PZ, SP.

## Funding

The work was supported by Ministero dell’ Università e della Ricerca (Progetti di Rilevante Interesse Nazionale (PRIN), 2017, CODICE 2017PHRC8X_003) (to SP).

## Conflict of Interest

The authors declare that the research was conducted in the absence of any commercial or financial relationships that could be construed as a potential conflict of interest.
