# Supplementary material for: Peritumoral Immune Infiltrate as a Prognostic Biomarker in Thin Melanoma
Source: Front Immunol. 2020 Sep 29;11:561390. doi: 10.3389/fimmu.2020.561390 (PMC7550791; doi:10.3389/fimmu.2020.561390)
Supplement: Supplementary file 1 [file Table_1.docx]

**Supplementary DATA**

**
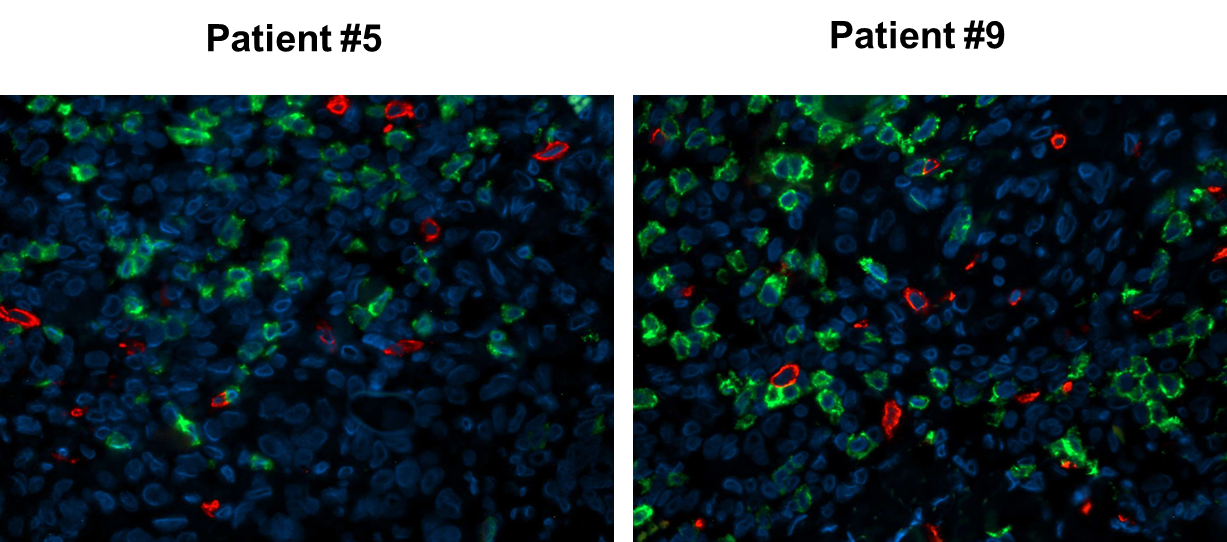
**

Double-fluorescence staining of CD8 and GRZ-B were conducted on a total of 2 representative FFPE tissue sections from thin melanoma tumor samples with a high number of CD8+ and GRZ-B+ infiltrating cells at IHC staining. Following their deparaffinization and hydration, prepared slides were incubated with antigen retrieval in a pressure cooker (Biocare) for 10 min at 110 °C. Following an incubation with protein blocking, slides were incubated with a cocktail of CD8-speficic mAb C8/144B and GRZ-B-specific mAb ab4059 at RT. Primary antibodies were detected utilizing a cocktail of goat anti-mouse IgG dylight 488 (green) and goat anti-rabbit IgG dylight 594 (red). After washing for two times, nuclei were stained with DAPI at room temperature for 10 min, and stored at 4 °C. The slides were examined using a fluorescent microscope (Olympus BX61).
